# Pathogenesis, Diagnosis, and Treatment of Hemostatic Disorders in COVID-19 Patients

**DOI:** 10.32607/actanaturae.11182

**Published:** 2021

**Authors:** A. F. Khalirakhmanov, K. F. Idrisova, R. F. Gaifullina, S. V. Zinchenko, R. I. Litvinov, A. Z. Sharafeev, A. P. Kiyasov, A. A. Rizvanov

**Affiliations:** University Hospital “Kazan Federal University”, Kazan, 420043 Russia; Kazan Federal University, Kazan, 420012 Russia; Republican Clinical Hospital named after Sh.Sh. Ependiev, Grozniy, 364030 Russia; University of Pennsylvania Perelman School of Medicine, Philadelphia, PA, 19104-6058 USA

**Keywords:** coronavirus, hemostatic disorders, thrombosis, anticoagulants, cytokine storm, COVID-19

## Abstract

The novel coronavirus infection named COVID-19 was first detected in Wuhan, China, in December 2019, and it has been responsible for significant morbidity and mortality in scores of countries. At the time this article was being written, the number of infected and deceased patients continued to grow worldwide. Most patients with severe forms of the disease suffer from pneumonia and pulmonary insufficiency; in many cases, the disease is generalized and causes multiple organ failures and a dysfunction of physiological systems. One of the most serious and prognostically ominous complications from COVID-19 is coagulopathy, in particular, decompensated hypercoagulability with the risk of developing disseminated intravascular coagulation. In most cases, local and diffuse macro- and microthromboses are present, a condition which causes multiple-organ failure and thromboembolic complications. The causes and pathogenic mechanisms of coagulopathy in COVID-19 remain largely unclear, but they are associated with systemic inflammation, including the so-called cytokine storm. Despite the relatively short period of the ongoing pandemic, laboratory signs of serious hemostatic disorders have been identified and measures for specific prevention and correction of thrombosis have been developed. This review discusses the causes of COVID-19 coagulopathies and the associated complications, as well as possible approaches to their early diagnosis, prevention, and treatment.

## INTRODUCTION

Coronaviruses (CoVs) are large, pleomorphic, and unsegmented RNA viruses that are abundant in mammals, especially in humans [1, 2, 3]. To date, six types of human coronavirus have been identified (HCoV-229E, -OC43, -NL63, -HKU1, MERS-CoV, SARS-CoV). They can cause upper respiratory-tract infection of varying severity, including the severe acute respiratory syndrome (SARS) [3]. At the end of 2019, a novel coronavirus was isolated from the epithelial cells of the human respiratory tract, which was named severe acute respiratory syndrome-associated coronavirus 2 (SARS-CoV-2) [4].

From the moment the novel pneumonia, defined as coronavirus disease 2019 (COVID-19), started spreading in China and other countries, the number of patients worldwide has steadily increased, including patients with severe pneumonia [2]. COVID-19 can lead to critical condition, with an acute respiratory distress syndrome and multiple-organ failure, which are in many cases caused by systemic coagulopathy [5]. Patients with the viral infection can develop sepsis that causes disseminated intravascular coagulation (DIC) in 30–50% of cases [6]. The DIC syndrome is an acquired clinical-biological syndrome characterized by a systemic intravascular activation of coagulation, which is induced by various causes, and thrombosis in the microvasculature, leading to organ dysfunction [7]. Clinical variants of the DIC syndrome are diverse, and its pathogenesis is very complex and not yet fully understood. In particular, in the sepsis-associated DIC syndrome, monocytes and endothelial cells are activated, which is accompanied by the release of cytokines, expression of the tissue factor, and secretion of the von Willebrand factor. Massive thrombi formation leads to the consumption of fibrinogen, antithrombin III, and other blood coagulation factors, as well as to thrombocytopenia, which are collectively referred to as “consumption coagulopathy” and can manifest itself in the form of hemorrhagic diathesis. The later stages of the DIC syndrome are associated with fibrinolysis activation aimed at recanalization of blood vessels, which can aggravate bleeding. Typical laboratory signs of the DIC syndrome include hypofibrinogenemia, thrombocytopenia, antithrombin III deficiency, and prolonged clotting tests, in combination with the clinical picture of blood circulatory disorders. The typical features are increased levels of the D-dimer and fibrin degradation products (FDPs), which are markers of fibrin deposition and secondary fibrinolysis [8]. A number of studies have indicated that the DIC syndrome is characteristic of COVID-19 and is, especially, often associated with mortality; however, the bleeding component, unlike in septic DIC, is absent in COVID-19 [8].

There is a close relationship between hemostatic disorders and the systemic inflammatory response to viral infection [9]. Clinical and laboratory signs of thrombotic conditions and their severity correlate directly with the production of inflammatory cytokines such as IL-2, IL-6, IL-7, IL-10, G-CSF, IP10, MCP-1, MIP-1A, and TNF-α, although the causes and mechanisms of “cytokine storm” development in either COVID-19 or other viral infections are not yet fully understood [10]. The relationship between inflammation and thrombosis and the ability of these two processes to exacerbate each other have been described in many pathological conditions [11, 12]. Physiological pro- and anticoagulants, as well as platelets, have pro-inflammatory properties independent of their hemostatic functions [13, 14, 15, 16, 17]. The interdependence of thrombotic complications and the systemic inflammatory response is one of the main links in COVID-19 pathogenesis [18, 19, 20].

laboratory parameters of hemostasis in COVID-19 patients. According to the published data, routine laboratory tests enable the identification of threatening and existing hemostatic disorders and the development of adequate and relevant approaches to the prevention and treatment of hemostatic disorders in COVID-19 patients. All the data on coagulopathies in COVID-19 reported to date have been obtained in relatively small patient cohorts. The findings obtained at the peak of the pandemic are preliminary and require a careful retrospective analysis.

## COVID-19 AND BLOOD COAGULATION DISORDERS

A study by Guan *et al*., who reported data on 1,099 patients with a laboratory-confirmed COVID-19 infection, showed that blood D-dimer levels in COVID-19 patients were significantly higher than the normal values and were consistent with high levels of the C-reactive protein. In severe cases, deviations of laboratory parameters (leukopenia, lymphopenia, thrombocytopenia) were more pronounced than those in mild symptoms of the disease [20].

Researchers from a Chinese clinical hospital examined 94 patients diagnosed with COVID-19 and 40 individuals in the control group, in accordance with the “pneumonia diagnosis protocol for novel coronavirus infection” that included coagulation tests [21]. The coagulation tests included the following laboratory parameters: activated partial thromboplastin time (aPTT), antithrombin (AT), fibrinogen/fibrin degradation products (FDP), fibrinogen, prothrombin time (PT), international normalized ratio (INR), thrombin time (TT), and D-dimer. Then, the COVID-19 patients were divided into three subgroups with mild, severe, and critical clinical symptoms of the disease, respectively. No significant differences in aPTT, PT, and INR were found between the three subgroups and the control group. The antithrombin value in all three subgroups was lower than that in the control group, but there was no difference among the COVID-19 subgroups. The blood D-dimer level in the patients with severe symptoms was significantly higher than that in the control group [21]. Tang *et al. *conducted an analysis of coagulation tests in 183 COVID-19 patients. It revealed that the D-dimer value in patients with severe symptoms who died was almost 3.5-fold higher, on average, than the normal values. The FDP, PT, and aPTT values were also higher than those in the survived patients. These results showed that the coagulation parameters in the deceased patients were similar to those in the DIC syndrome [8]. Thus, excessive activation of blood coagulation leads to the development of the DIC syndrome, which is an unfavorable prognostic factor in COVID-19 [22].

The D-dimer is a product of fibrinolytic degradation of fibrin cross-linked by factor XIIIa; therefore, an increase in the blood D-dimer concentration is used in clinical laboratory diagnostics of micro- and macrothrombosis [23]. Examination of 191 COVID-19 patients showed that D-dimer values in non-survived patients were almost 9-fold higher [24]. Clinical data, laboratory parameters, and results of chest-computed tomography of 248 COVID-19 patients were retrospectively analyzed. The D-dimer level was high (≥ 0.5 mg/L) in 75% of the patients. In hospitalized patients, the D-dimer level climbed significantly as the severity of COVID-19 increased. In moderately severe patients, the median level of D-dimer was approximately 7-fold higher than the normal values and increased to critical values in severe patients. Other researchers have also identified changes in hemostasis; in particular, an increase in the blood D-dimer level in COVID-19 patients [25, 26]. Higher D-dimer levels are found in patients with concomitant critical diseases (chronic heart failure, respiratory diseases, malignant neoplasms, etc.); therefore, the D-dimer level may be used as a prognostic marker of mortality in COVID-19 [27].

The clinical and laboratory data of 41 patients hospitalized with a confirmed diagnosis of COVID-19 were reported. Higher PT values and D-dimer levels were noted in patients requiring transfer to an intensive care unit [28].

Zhang *et al*. reported three COVID-19 cases with severe pneumonia and coagulopathy. All the patients had a hypertension history; two patients had a coronary heart disease; one patient had a stroke. On examination, there were signs of ischemia in the lower extremities on both sides. Laboratory tests showed increased PT, aPTT, fibrinogen, and D-dimer levels, leukocytosis, and thrombocytopenia [29]. The presence of antiphospholipid antibodies in the blood indicates development of the antiphospholipid syndrome; however, these antibodies can be temporarily produced in patients with various infections [30]. The presence of these antibodies can lead to thrombotic complications that, in critical patients, are difficult to differentiate from other types of diffuse microthrombosis, such as DIC, heparin-induced thrombocytopenia, and thrombotic microangiopathy.

Therefore, COVID-19 is associated with pronounced changes in the laboratory parameters of hemostasis; an elevated D-dimer level (≥ 1 μg/mL) is considered an unfavorable prognostic factor [24, 31, 32, 33].

## COVID-19 AND THROMBOCYTOPENIA

A meta-analysis by Lippi *et al*. revealed a decrease in the platelet count in patients with severe COVID-19 (mean 31 × 109/L, 95% CI: 29 × 10^9^ to 35 × 109/L), with thrombocytopenia being associated with a five-fold increase in the risk of a severe form of the disease [34]. Thrombocytopenia often occurs in patients with a critical course of the disease and is usually combined with multiple-organ pathology and coagulopathy in the form of the DIC syndrome [35]. Thrombocytopenia, which is considered a mortality risk factor, was found in 55% of the patients with the severe acute respiratory syndrome [36].

Along with consumption of platelets for the formation of thrombi, thrombocytopenia in COVID-19 is associated with the ability of the coronavirus to directly affect the bone marrow, which leads to abnormal hematopoiesis or triggers an autoimmune response to hematopoietic and stromal bone marrow cells [36, 37]. The platelet count in COVID-19 is a simple and readily available biomarker associated with the clinical picture and mortality risk [38, 39]. It should be noted that a low blood platelet count correlates with elevated indicators of disease severity and multiple organ dysfunction, such as the New Simplified Acute Physiology Score II (SAPS II) and Acute Physiology and Chronic Health Evaluation II (APACHE II) [39].

## “CYTOKINE STORM” IN COVID-19

There is growing evidence of “cytokine storm” development in severe COVID-19 [40], as a response to systemic inflammation [9]. Inflammation is an integral part of an effective immune response, which enables the neutralization and elimination of the infectious agent. Massive formation of inflammatory cytokines accompanies a pronounced inflammation and leads to a high permeability of blood vessels, multiple-organ failure, and, probably, death at very high blood cytokine concentrations [41]. The term “cytokine storm” in relation to infectious diseases was introduced for the first time in the early 2000s during a study of the cytomegalovirus infection [42], Epstein-Barr virus-associated hemophagocytic lymphohistiocytosis [43], group A streptococcus [44], influenza virus [45], hantavirus [46], variola virus [47], and the severe acute respiratory syndrome coronavirus (SARS-CoV) [48].

Cytokines are a diverse group of small proteins that are secreted by cells for intercellular communication [49]. A complex cytokine response is considered as a series of overlapping reactions, each with its own degree of redundancy and alternative pathway. This combination of overlap and redundancy is important in identifying key steps in the cytokine response to the infection and in identifying specific cytokines for therapeutic intervention.

There have been many studies in humans and experimental models that have convincingly proven the pathogenic role of inflammatory cytokines/chemokines derived from inflammatory monocyte-macrophages and neutrophils. The effect of coronavirus on cytokine production in the acute phase of the disease was characterized by measuring the levels of the plasma cytokines IL-1B, IL-1RA, IL-2, IL-4, IL-5, IL-6, IL-7, IL-8 (known as CXCL8), IL-9, IL-10, IL-12p70, IL-13, IL-15, IL-17A, eotaxin (known as CCL11), basic FGF2, G-CSF (CSF3), GM-CSF (CSF2), IFN-γ, IP10 (CXCL10), MCP-1 (CCL2), MIP-1A (CCL3), MIP-1B (CCL4), PDGFB, RANTES (CCL5), TNF-α, and VEGFA [28]. Critical care patients were found to have higher plasma levels of IL-2, IL-7, IL-10, G-SCF, IP10, MCP-1, MIP1-A, and TNF-α. These findings suggest that the “cytokine storm” is associated with a severity of the disease [28]. Therefore, therapeutic interventions targeting pro-inflammatory cytokines can attenuate excessive inflammatory responses. It is also important to note that high viral titers at the early and later stages of the infection are strongly correlated with the severity of the disease. Therefore, strategies aimed at controlling the viral load and attenuating the inflammatory responses are very important in the treatment and management of patients. This approach requires more research to identify the specific signaling pathways that mediate inflammatory responses in coronavirus patients [50].

## OTHER HEMATOLOGICAL CHANGES IN COVID-19

The most common hematologic findings include lymphocytopenia [51–53], neutrophilia [54], eosinopenia [55], mild thrombocytopenia [53], and, less commonly, thrombocytosis [34]. The leukocyte counts can be normal, decreased [28], or increased [24]. According to a meta-analysis [56], leukocytosis, lymphopenia, and thrombocytopenia in a COVID-19 infection are associated with a more severe course of the disease and even death. According to Terpos *et al*., during the first days of the disease, when non-specific symptoms are present, the leukocyte and lymphocyte counts are normal or slightly reduced [57]. Later, on days 7–14 of the infection, the disease affects organs with a higher expression of the angiotensin-converting enzyme 2 (ACE2) [58], a SARS-CoV-2 receptor, such as the lungs, heart, and gastrointestinal tract. At this stage, more pronounced hematological changes, in particular a significant decrease in the lymphocyte count, are present. This is more typical of non-survived patients. In survived patients, the lowest lymphocyte count was encountered around day 7 of symptoms onset, followed by recovery [24]. Thus, the dynamics of the lymphocyte count, i.e. their serial count over time, may be a predictor of the disease’s clinical outcome. An analysis of the published data showed that, among all hematological changes, lymphopenia is one of the most frequent indicators of a lethal outcome. Ratios of blood cell counts are of great clinical importance: e.g., a reduced lymphocyte/leukocyte ratio indicates severe symptoms and/or a lethal outcome [59]. Similarly, increased neutrophil/lymphocyte and neutrophil/ platelet ratios may indicate myocardial damage and increased mortality [60]. Therefore, it is important to monitor hematological parameters to assess COVID-19 progression and prognosis.

## PROPHYLAXIS AND TREATMENT OF COAGULOPATHY IN COVID-19

A high rate of thrombotic complications has spurred interest in thromboprophylaxis and anticoagulant therapy in COVID-19. Data on systemic hypercoagulability, in particular massive thrombinemia and diffuse microthrombosis accompanied by multiple organ failures, are used as a pathogenic rationale for treatment. Therefore, inhibition of thrombin formation and/or activity in the blood may potentially decrease the risk and prevalence of thrombosis and reduce mortality in COVID-19 [23, 37].

The most common method for the prophylaxis and treatment of thrombosis in COVID-19 patients is the use of low-molecular-weight heparin (LMWH) [61]. LMWH should be administered to all patients (including non-critical ones) who require hospitalization for COVID-19 in the absence of contraindications (active bleeding and a platelet count of less than 25 × 109/L). The efficacy of prophylactic heparin therapy was shown in a study of 449 patients with severe COVID-19; of those, 99 patients received heparin (mainly LMWH) at prophylactic doses [62]. Although there were no differences in the 28-day mortality in patients untreated and treated with heparin, LMWH in patients with more pronounced hemostatic disorders (sepsis-induced coagulopathy score ≥ 4) reduced significantly the mortality rate (40% versus 64%, *p *= 0.029). Heparin therapy reduced mortality in patients with a 6-fold or more elevated level of D-dimer (33% versus 52%, *p *= 0.017) [62]. In addition, LMWH administration reduced the risk of pulmonary embolism in critical patients.

The possible effect of other drugs received by patients should be considered when evaluating the dose of LMWH. Approximately 50% of the patients who died from COVID-19 in Italy had multiple comorbidities, such as atrial fibrillation or coronary heart disease, which required anticoagulant or antiplatelet treatment. The treatment of these patients is particularly challenging due to potential interactions between heparin and other drugs, such as new oral anticoagulants [63] that have proven themselves well in the prophylaxis and treatment of venous thromboembolism; these drugs may also be promising for reducing the risk of thrombosisin in COVID-19 patients [41].

## CONCLUSIONS

COVID-19 patients often develop hemostatic disorders: in particular, hypercoagulability of varying severity. Typical laboratory signs of these disorders are thrombocytopenia, increased D-dimer and fibrinogen concentrations in the blood, and prolonged PT and aPTT, especially in patients with severe COVID-19. Dynamic monitoring of these hemostatic parameters may reflect a transformation of the clinical course of the disease into a more severe case. The most pronounced changes in hemostasis in COVID-19 have an unfavorable prognostic value. Given the increased risk of thromboembolic complications in COVID-19 patients, prophylactic and therapeutic use of anticoagulants, primarily low-molecular-weight heparins, is justified.

## Abbreviations

COVID-19: coronavirus disease 2019
SARS-CoV-2: severe acute respiratory syndrome coronavirus 2
DIC: disseminated intravascular coagulation
IL: interleukin
G-CSF: granulocyte colony-stimulating factor
MCP-1: monocyte chemotactic factor-1
TNF-α: tumor necrosis factor-α
aPTT: activated partial thromboplastin time
AT: antithrombin
FDP: fibrinogen/fibrin degradation product
PT: prothrombin time
INR: international normalized ratio
TT: thrombin time
LMWH: low molecular weight heparin
NOAC: novel oral anticoagulant
PE: pulmonary embolism.

## References

[1] Woo P.C.Y., Huang Y., Lau S.K.P., Yuen K.Y. (2010). Viruses..

[2] Cui J., Li F., Shi Z.L. (2019). Nat. Rev. Microbiol..

[3] Nikiforov V.V., Suranova T.G., Chernobrovkina T.Yu., Yankovskaya Y.D., Burova S.V. (2020). Russ. Arch. Internal Med..

[4] Zhu N., Zhang D., Wang W., Li X., Yang B., Song J., Zhao X., Huang B., Shi W., Lu R. (2020). N. Eng. J. Med..

[5] Mattiuzzi C., Lippi G. (2020). Ann. Tansl. Med..

[6] Costello R.A., Nehring S.M. (2020). Treasure Island, FL: Stat Pearls Publ..

[7] Taylor F.B., Toh C.H., Hoots K.W., Wada H., Levi M. (2001). Thromb. Haemostasis..

[8] Tang N., Li D., Wang X., Sun Z. (2020). J. Thromb. Haemost..

[9] Scharrer I. (2018). Front. Biosci..

[10] Sarzi-Puttini P., Giorgi V., Sirotti S., Marotto D., Ardizzone S., Rizzardini G., Antinori S., Galli M. (2020). Clin. Exp. Rheumatol..

[11] Iba T., Levy J.H. (2018). J. Thromb. Haemost..

[12] Jackson S.P., Darbousset R., Schoenwaelder S.M. (2019). Blood..

[13] Claushuis T.A., de Stoppelaar S.F., Stroo I., Roelofs J.J., Ottenhoff R., van der Poll T., van ‘t Veer C. (2017). J. Thromb. Haemost..

[14] Chen J., Li X., Li L., Zhang T., Zhang Q., Wu F., Wang D., Hu H., Tian C., Liao D., Zhao L. (2019). Cell Res..

[15] Burzynski L.C., Humphry M., Pyrillou K., Wiggins K.A., Chan J.N., Figg N., Kitt L.L., Summers C., Tatham K.C., Martin P.B. (2019). Immunity..

[16] Vardon-Bounes F., Ruiz S., Gratacap M.P., Garcia C., Payrastre B., Minville V. (2019). Int. J. Mol. Sci..

[17] Assinger A., Schrottmaier W.C., Salzmann M., Rayes. J. (2019). Front. Immunol..

[18] Delvaeye M., Conway E.M. (2009). Blood..

[19] Giannis D., Ziogas I. A., Gianni P. (2020). J. Clin. Virol..

[20] Guan W.J., Ni Z.Y., Hu Y., Liang W.H., Ou C.Q., He J.X., Liu L., Shan H., Lei C.., Hui D.S.C. (2020). N. Engl. J. Med..

[21] Han H., Yang L., Liu R., Liu F., Wu K.L., Li J., Liu X.H., Zhu C.L. (2020). Clin. Chem. Lab. Med..

[22] Kawano N., Wada H., Uchiyama T., Kawasugi T., Madoiwa S., Takezako N., Suzuki K., Seki Y., Ikezoe T., Hattori T., Okamoto K. (2020). Thrombosis J..

[23] Schutte T., Thijs A., Smulders Y.M. (2016). Neth. J. Med..

[24] Zhou F., Yu T., Du R., Fan G., Liu Y., Liu Z., Xiang J., Wang Y., Song B., Gu X. (2020). Lancet..

[25] Chen N., Zhou M., Dong X., Qu J., Gong F., Han Y., Qiu Y., Wang J., Liu Y., Wei Y. (2020). Lancet..

[26] Wu J., Liu J., Zhao X., Liu C., Wang W., Wang D., Xu W., Zhang C., Yu J., Jiang B., Cao H., Li L. (2020). Clin. Infect. Dis..

[27] Yumeng Y., Cao J., Wang Q., Liu K., Luo Z., Yu K., Chen X., Hu B., Huang Z. (2020). Crit. Care Med..

[28] Huang C., Wang Y., Li X., Ren L., Zhao J., Hu Y., Zhang L., Fan G., Xu J., Gu X. (2020). Lancet..

[29] Zhang Y., Xiao M., Zhang S., Xia P., Cao W., Jiang W., Chen H., Ding X., Zhao H., Zhang H. (2020). N. Engl. J. Med..

[30] Uthman I.W., Gharavi A.E. (2002). Semin. Arthritis Rheum..

[31] Querol-Ribelles J. M., Tenias J.M., Grau E., Querol-Borras J.M., Climent J.L., Gomez E., Martinez I. (2004). Chest..

[32] Fruchter O., Yigla M., Kramer M. R. (2015). Am. J. Med. Sci..

[33] Snijders D., Schoorl M., Schoorl M., Bartels P.C., van der Werf T.S., Bo-ersma W.G. (2012). Eur. J. Case Rep. Intern. Med..

[34] Lippi G., Mario P., Brandon M.H. (2020). Clin. Chim. Acta..

[35] Zarychanski R., Houston D. S. (2017). Hematol. Am. Soc. He matol. Edu. Program..

[36] Yang M., Ng M.H., Li C.K. (2005). Hematology..

[37] Jolicoeur P., Lamontagne L. (1995). Adv. Exp. Med. Biol..

[38] Khurana D., Deoke S.A. (2017). Indian J. Crit. Care Med..

[39] Vanderschueren S., De Weerdt A., Malbrain M., Vankersschaever D., Frans E., Wilmer A., Bobbaers H. (2000). Crit. Care Med..

[40] Mehta P., McAuley D.F., Brown M., Sanchez E., Tattersall R.S., Manson J.J. (2020). Lancet..

[41] Jose R.J., Manuel A. (2020). Lancet Respir. Med..

[42] Barry S.M., Johnson M.A., Janossy G. (2000). Bone Marrow Transplant..

[43] Imashuku S. (2002). Crit. Rev. Oncol. Hematol..

[44] Bisno A.L., Brito M.O., Collins C.M. (2003). Lancet Infect. Dis..

[45] Yokota S. (2003). Nihon Rinsho..

[46] Garanina E., Martynova E., Davidyuk Y., Kabwe E., Ivanov K., Titova A., Markelova M., Zhuravleva M., Cherepnev G., Shakirova V.G. (2019). Viruses..

[47] Jahrling P.B., Hensley L.E., Martinez M.J., LeDuc J.W., Rubins K.H., Relman D.A., Huggins J.W. (2004). Proc. Natl. Acad. Sci. USA..

[48] Huang K.J., Su I.J., Theron M., Wu Y.C., Lai S.K., Liu C.C., Lei H.Y. (2005). J. Med. Virol..

[49] Tisoncik J.R., Korth M.J., Simmons C.P., Farrar J., Martin T.R., Katze M.G. (2012). Microbiol. Mol. Biol. Rev..

[50] Channappanavar R., Perlman S. (2017). Semin. Immunopathol..

[51] Ruan Q., Yang K., Wang W., Jiang L., Song J.C. (2020). Intensive Care Med..

[52] Wang F., Nie J., Wang H., Zhao Q., Xiong Y., Deng L., Song S., Ma Z., Mo P., Zhang Y. (2020). J. Infect. Dis..

[53] Sun S., Cai X., Wang H., He G., Lin Y., Lu B., Chen C., Pan Y., Hu X. (2020). Clin. Chim. Acta..

[54] Qian G.Q., Yang N.B., Ding F., Ma A.H.Y., Wang Z.Y., Shen Y.F., Shi C.W., Lian X., Chu J.G., Chen L. (2020). QJM: An International Journal of Medicine..

[55] Liu F., Xu A., Zhang Y., Xuan W., Yan T., Pan K., Yu W., Zhang J. (2020). Int. J. Infect. Dis..

[56] Henry B.M., de Oliveira M.H.S., Benoit S., Plebani M., Lippi G. (2020). Clin. Chem. Lab. Med..

[57] Terpos E., Ntanasis-Stathopoulos I., Elalamy I., Kastritis E., Sergentanis T.N., Politou M., Psaltopoulou T., Gerotziafas G., Dimopoulos M.A. (2020). Am. J. Hematol..

[58] Zhou P., Yang X.L., Wang X.G., Hu B., Zhang L., Zhang W., Si H.R., Zhu Y., Li B., Huang C.L. (2020). Nature.

[59] Qin C., Zhou L., Hu Z., Zhang S., Yang S., Tao Y., Xie C., Ma K., Shang K., Wang W. (2020). Clin. Infect. Dis..

[60] Guo T., Fan Y., Chen M., Wu X., Zhang L., He T., Wang H., Wan J., Wang X., Lu Z. (2020). JAMA Cardiol..

[61] Song J.C., Wang G., Zhang W., Zhang Y., Li W.Q., Zhou Z. (2020). Mil. Med. Res..

[62] Tang N., Bai H., Chen X., Gong J., Li D., Sun Z. (2020). J. Thromb. Haemost..

[63] Marietta M., Ageno W., Artoni A., De Candia E., Gresele P., Marchetti M., Marcucci R., Tripodi A. (2020). Blood Transfus..

